# Beware the algorithm

**DOI:** 10.7554/eLife.69657

**Published:** 2021-05-26

**Authors:** Peter van Endert

**Affiliations:** Institut National de la Santé et de la Recherche Médicale, Unité 1151, Université de Paris, Centre National de la Recherche Scientifique, UMR 8253ParisFrance

**Keywords:** TCR gene therapy, proteasome processing, spliced epitopes, cancer, Human, Mouse

## Abstract

Spliced peptides present on tumor cells can help to mount an immune response, but algorithms offer limited help in predicting which ones actually exist and perform this role in vivo.

**Related research article** Willimsky G, Beier C, Immisch L, Papafotiou G, Scheuplein V, Goede A, Holzhütter HG, Blankenstein T, Kloetzel PM. 2021. In vitro proteasome processing of neo-splicetopes does not predict their presentation in vivo. *eLife* **10**:e62019. doi: 10.7554/eLife.62019

The human immune system is a formidable surveillance system that helps to keep cancers in check. Killer T cells, for example, can spot and deactivate tumors: more precisely, they can recognize short peptides which are displayed on the surface of harmful cells by a group of molecules called human leukocyte antigens or HLA (Klein and Sato, 2000). Many variations of the HLA genes exist, each coding for a slightly different molecule that can only bind to a limited set of peptides. In turn, these peptides are created inside target cells through a complex protein degradation process supported by a large enzyme known as the proteasome (Rock et al., 2010). For killer T cells to specifically deactivate tumors, cancer cells should be carrying at least one type of HLA molecule that can bind to peptides produced exclusively or primarily in these diseased cells. It is very rare, however, to find a peptide that is only present on tumors.

One way to overcome this obstacle is to focus on the altered peptides produced by driver mutations in genes that regulate cell growth, and are therefore often changed in cancer (Blankenstein et al., 2015). Algorithms could help in that search. These computer-implementable instructions are developed using existing data to ‘automatically’ predict the outcomes of complex biological processes, such as which peptides could be generated by the protein degradation process. Yet algorithms are never failsafe, and they can even be treacherous when fed sketchy data. Now, in eLife, Gerald Willimsky, Peter Kloetzel and colleagues at the Charité hospital in Berlin and various German institutions report having experienced this the hard way (Willimsky et al., 2021).

The team was hunting peptides that could trigger or boost the activity of killer T cells against tumors, seeking to exploit the KRAS^G12V^ and RAC2^P29L^ driver mutations. But they found that the peptides coded by the mutated genes could not bind to HLA-A2, the most frequent HLA variant in Caucasians. This led the researchers to turn to a published algorithm that predicted the production of ‘spliced peptides’ that fit the HLA-A2 molecule (Mishto et al., 2019).

Peptide slicing is a fairly new and partly controversial concept in immunology. It proposes that the proteasome sometimes produces two peptides which can fuse, resulting in a ‘spliced peptide’ containing two fragments of the source protein but lacking several amino acids in-between (Vigneron et al., 2017). Solid data show that a small number of these peptides are actually produced in vitro, in isolated live cells, and in vivo: according to some authors, up to 25% of all proteins that bind to HLA molecules are thought to be spliced peptides – but this value could be much lower (Liepe et al., 2016; Mylonas et al., 2018). A small number of spliced peptides have been shown to activate specific killer cell responses in mouse models (Hanada et al., 2004; Warren et al., 2006).

When Willimsky et al. used the algorithm to predict which spliced peptides could match the HLA-A2 allele, several sequences were returned both for KRAS^G12V^ and RAC2^P29L^. This prompted the team to embark on a series of in vitro and in vivo experiments to check whether these peptides could actually bind to HLA-A2. And indeed, when mice that had been genetically modified to express human HLA-A2 were exposed to the peptides, this led to the production of killer T cells that could react to these sequences. Willimsky et al. then genetically modified certain human immune cells to express specific T cell receptors, and these could spot and kill HLA-A2-expressing cells that had been pre-incubated with the relevant peptides. Both mice and human killer cells were therefore perfectly able to respond to the mutant tumor peptides.

However, further in vitro experiments showed that proteasome digestions only produced the RAC2^P29L^ spliced peptide. More importantly, highly sensitive killer T cells were unable to recognize and deactivate tumor cell lines that expressed the mutant proteins, even when the cells overexpressed pieces of the mutant proteins containing the two fragments that fuse together to form the spliced peptide. This means that, in live cells, the splicing either did not happen or it did not create enough peptide to activate a response by the killer T cells (Figure 1).

**Figure 1.**
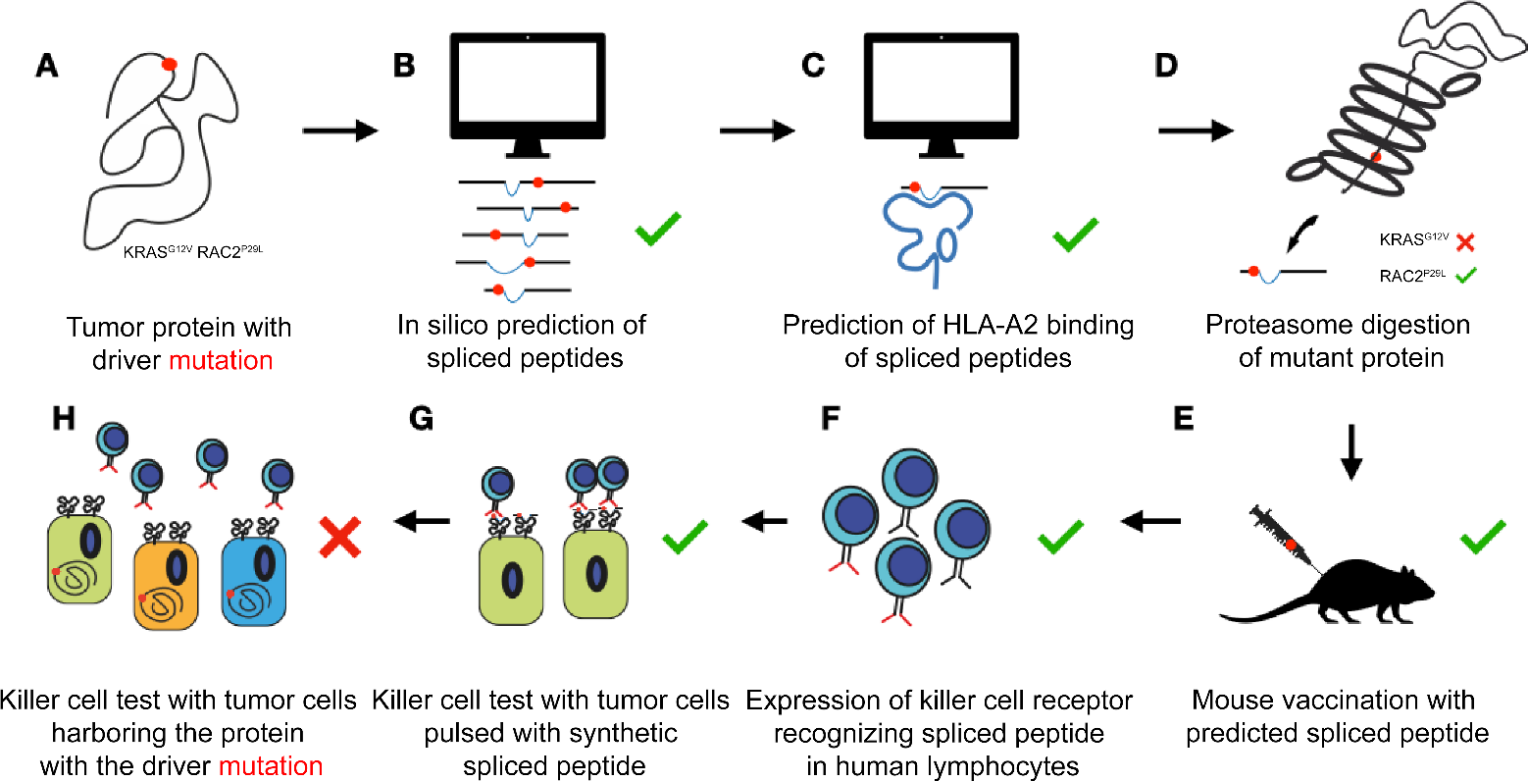
Algorithms poorly predict which spliced peptides can help the immune system recognize cancer cells. Two proteins that often carry a mutation (red dot) that drives cancer (KRAS^G12V^ and RAC2^P29L^) are chosen for further exploration (**A**). An algorithm predicts multiple potential spliced peptides encompassing the mutations for each protein (**B**). A second algorithm identifies a small number of putative spliced peptides predicted to bind to HLA-A2 on the surface of target cells (**C**). In vitro, the proteasome does actually generate a predicted spliced peptide carrying the mutation for RAC2 but not for KRAS (**D**). Exposing mice to the predicted spliced peptides generates killer T cells that identify the peptides with high affinity (**E**). The T cell receptors that bind to the spliced peptides are successfully transferred to human immune cells called lymphocytes (**F**). These ‘transformed’ cells efficiently recognize tumor cells pulsed with the synthetic spliced peptides (**G**). However, different tumor cell lines that express the mutant proteins (but are not artificially equipped with the spliced peptides) are not recognized by the transformed human immune cells. This suggests that, despite the algorithm’s prediction, these peptides are not produced (or are not produced in large enough numbers) in actual cells (**H**).

What can be learnt from what Willimsky et al. certainly considered a setback? These results could be dismissed simply as bad luck: after all, the non-spliced peptides predicted by an algorithm also are not fully foolproof. Even without considering peptide splicing, the outcome of protein degradation in cells is notoriously difficult to predict. In future research, it is certainly sensible to test early on whether predicted spliced peptides are actually produced in live cells.

Nevertheless, it is likely that using algorithms to predict spliced peptides production is still premature. There is still a lack of high quality data which verify that these putative sequences are indeed produced in vitro under physiologic conditions, as well as in live cells. These studies are sorely needed to improve future algorithms and find new targets for cancer treatment.

## References

[bib1] Blankenstein T, Leisegang M, Uckert W, Schreiber H (2015). Targeting cancer-specific mutations by T cell receptor gene therapy. Current Opinion in Immunology.

[bib2] Hanada K, Yewdell JW, Yang JC (2004). Immune recognition of a human renal cancer antigen through post-translational protein splicing. Nature.

[bib3] Klein J, Sato A (2000). The HLA system. New England Journal of Medicine.

[bib4] Liepe J, Marino F, Sidney J, Jeko A, Bunting DE, Sette A, Kloetzel PM, Stumpf MP, Heck AJ, Mishto M (2016). A large fraction of HLA class I ligands are proteasome-generated spliced peptides. Science.

[bib5] Mishto M, Mansurkhodzhaev A, Ying G, Bitra A, Cordfunke RA, Henze S, Paul D, Sidney J, Urlaub H, Neefjes J, Sette A, Zajonc DM, Liepe J (2019). An in silico-in vitro pipeline identifying an HLA-A^*^02:01^+^ KRAS G12V^+^ spliced epitope candidate for a broad tumor-immune response in cancer patients. Frontiers in Immunology.

[bib6] Mylonas R, Beer I, Iseli C, Chong C, Pak HS, Gfeller D, Coukos G, Xenarios I, Müller M, Bassani-Sternberg M (2018). Estimating the contribution of proteasomal spliced peptides to the HLA-I ligandome. Molecular & Cellular Proteomics.

[bib7] Rock KL, Farfán-Arribas DJ, Shen L (2010). Proteases in MHC class I presentation and cross-presentation. The Journal of Immunology.

[bib8] Vigneron N, Ferrari V, Stroobant V, Abi Habib J, Van den Eynde BJ (2017). Peptide splicing by the proteasome. Journal of Biological Chemistry.

[bib9] Warren EH, Vigneron NJ, Gavin MA, Coulie PG, Stroobant V, Dalet A, Tykodi SS, Xuereb SM, Mito JK, Riddell SR, Van den Eynde BJ (2006). An antigen produced by splicing of noncontiguous peptides in the reverse order. Science.

[bib10] Willimsky G, Beier C, Immisch L, Papafotiou G, Scheuplein V, Goede A, Holzhütter HG, Blankenstein T, Kloetzel PM (2021). In vitro proteasome processing of neo-splicetopes does not predict their presentation in vivo. eLife.

